# Prevalence of sexual violence in Ethiopian workplaces: systematic review and meta-analysis

**DOI:** 10.1186/s12978-020-01050-2

**Published:** 2020-12-09

**Authors:** Mulugeta Dile Worke, Zewdie Birhanu Koricha, Gurmesa Tura Debelew

**Affiliations:** 1 Department of Midwifery, College of Health Sciences, Debre Tabor University, Debre Tabor, Ethiopia; 2 Department of Health, Behavior, and Society, Faculty of Public Health, Jimma University, Jimma, Ethiopia; 3 Department of Population and Family Health, Faculty of Public Health, Jimma University, Jimma, Ethiopia

**Keywords:** Workplace sexual violence, Cross-sectional studies, Pooled prevalence, Ethiopia, Systematic review, Meta-analysis

## Abstract

**Background:**

Workplace sexual violence is a significant public health problem in low and middle-income countries, including Ethiopia. However, except for individual studies with varying prevalence rates in different occupations, there are no national prevalence studies conducted in workplace settings in Ethiopia. Appropriate estimation of the problem is essential to formulate health service plans most fitted for workplaces. Hence, this review and meta-analysis intended to estimate the national pooled prevalence of workplace sexual violence in Ethiopia.

**Methods:**

The databases used were; PubMed, Google Scholar, CINAHL, and African Journals Online. For a critical appraisal of the papers, we used the Joanna Briggs Institute Meta-Analysis of Statistics Assessment and Review Instrument for cross-sectional studies. The meta-analysis was conducted using comprehensive meta-analysis and MetaXL software. Descriptive information of studies was presented in narrative form, and quantitative results were presented in forest plots. The Cochran Q test and I^2^ test statistics were employed to test heterogeneity across studies. A random-effect model computed the pooled estimate prevalence with 95% confidence intervals.

**Results:**

The pooled prevalence of workplace sexual violence was 22% (95% CI 17%, 28%). The pooled prevalence was 14.1% (95% CI 10.4%, 18.9%) for attempted rape, 8% (95% CI 5.7%, 11.1%) for rape, and 33.2% (95% CI 24.1%, 43.7%) for sexual harassment. The pooled prevalence was the highest among female university staffs 49% (95% CI 45%, 56%), and among commercial sex workers 28% (95% CI 3%, 59%).

**Conclusions:**

This analysis revealed that the prevalence of sexual violence and sexual harassment in Ethiopian workplaces is high. It was also exceptionally high among female faculty staff, commercial sex workers, and workplaces in Tigray National regional state. Thus, concerned stakeholders must design and implement effective interventions to prevent workplace sexual violence in workplaces in Ethiopia and provide necessary support and care to the victims.

## Plain English summary

Workplace sexual violence (WSV) results in negative organizational culture, long-term health and psychological impacts on employees, damage to research integrity, and a costly loss of skilled workforce. It mainly affects workers in the most vulnerable work situations who have poor access to labor rights such as freedom of association, collective bargaining, decent work, non-discrimination, and access to justice. It also increases the likelihood of sexually transmitted infections, unintended pregnancies, and unsafe abortion. In Ethiopia, workplace sexual violence is one of the high burdens of sexual and reproductive health problems. The prevalence of workplace sexual violence varies from workplace to workplace and from occupation to occupation in Ethiopia. Poor knowledge of the impacts of workplace sexual violence prevention mechanisms, transactional sex practices, and poverty are the major contributors to WSV.

Recently, WSV is a significant public health and human right agenda in Ethiopia. However, a lack of national summarized data could be one reason for the poor implementation of sexual violence prevention and control programs in workplaces. Except for individual studies with varying prevalence rates, there are no national prevalence studies conducted in workplace settings in Ethiopia. Appropriate estimates of the problem are essential to formulate health service plans most fitted for workplace settings. The researchers investigated the prevalence of workplace sexual violence in different workplaces, and these studies were highly scattered by profession, geography, and occupation. These results indicate that individual studies should be reviewed and summarized for better utilization. Our systematic review and meta-analysis intended to fill this gap by calculating the pooled prevalence of workplace sexual violence in Ethiopian workplaces. We included 31 articles with 21,054 participants. Our systematic review and meta-analysis revealed that workplace sexual violence is a common public health problem in Ethiopia. In this systematic review and meta-analysis, 22% of the participants were workplace sexual violence victims. Of the types of WSV, workplace sexual harassment is high in Ethiopia. It is also exceptionally high among female university staff, commercial sex workers, and workplace in Tigray National Regional States. Thus, as noted above, governmental organizations and other relevant stakeholders should develop effective programs and interventions to reduce workplace sexual violence prevalence over different Ethiopian workplaces and protect victims with relevant legislation.

## Background

Sexual violence (SV) is defined as “any sexual act, attempt to attain a sexual act, unwanted sexual comments or advances, or acts to traffic, or otherwise directed against, women’s sexuality, using coercion (i.e., psychological intimidation, physical force, or threats of harm), by any person regardless of connection to the target, in any setting, including but not limited to home and work [1, 2].” It is a societal issue that requires systemic change and is influenced by our larger social systems, including the workplace [3].

Workplace sexual violence (WSV) is part of workplace violence that takes verbal, non-verbal, and physical forms. It can be construed as unwanted, unreciprocated, or unwelcome behavior of a sexual nature tending to humiliate, threaten, or embarrass [4]. It includes sexual harassment, rape, and attempted rape [5]. Studies showed that women suffer physical, mental, and reproductive health consequences of sexual violence like depression, loss of self-confidence, injuries, unwanted pregnancy, sexually transmitted diseases, and disability up to death [5, 6]. Another study indicated that rape alone results in about 32,000 unwanted pregnancies each year globally [7]. The problem can have an emotional impact and is linked to adverse health behaviors, such as substance use and mood disorders like anxiety and depression [8, 9].

The WSV mainly affects workers in the most vulnerable work situations who have poor access to labor rights such as freedom of association, collective bargaining, decent work, non-discrimination, and access to justice [10]. Although everyone has the right to live and work free from violence, sexual violence in the world of work exists in all occupations and sectors of the economy globally [11]. However, despite tremendous efforts, 1 in 3 (35%) women are experiencing either physical or sexual violence, and 1 in 5 (20%) experience rape or attempted rape worldwide in 2006 [12]. It is also one of the social inequalities across a broad range of cases and contexts. The power of WSV lies both in its ubiquity as a tool of domination and the ease with which it is rendered invisible [13].

Moreover, WSV has devastating effects on victims’ health and well-being of victims and severe effects for the business owners and society [11]. It can fuel negative organizational culture [14]; it can result in long-term health and psychological impacts on employees and significant damage to research integrity, and a costly loss of skilled workforce in these fields [15]. Consequently, sustainable development goal five aims to achieve gender equality and empower all women and girls to encompass the elimination of all forms of violence against women and girls, and goal eight aims at full and productive employment and decent work for all [16]. However, there was uneven progress on gender equality, including decent work and freedom from violence [11].

Workplace sexual violence is a significant public health and human right agenda in Ethiopia [5]. However, a lack of national summarized data could be one reason for the poor implementation of sexual violence prevention and control programs in workplaces. Except for individual studies with varying prevalence rates, there are no national prevalence studies conducted in workplace settings in Ethiopia. Appropriate estimates of the problem are essential to formulate health service plans most fitted for workplace settings. The researchers investigated the prevalence of workplace sexual violence in different workplaces, and these studies were highly scattered by profession, geography, and occupation. These results indicate that individual studies should be reviewed and summarized for better utilization. Thus, this systematic review and meta-analysis intended to fill this gap by estimating the pooled prevalence of workplace sexual violence in workplaces in Ethiopia.

## Methods

### Data sources and search strategy

**Fig. 1:**
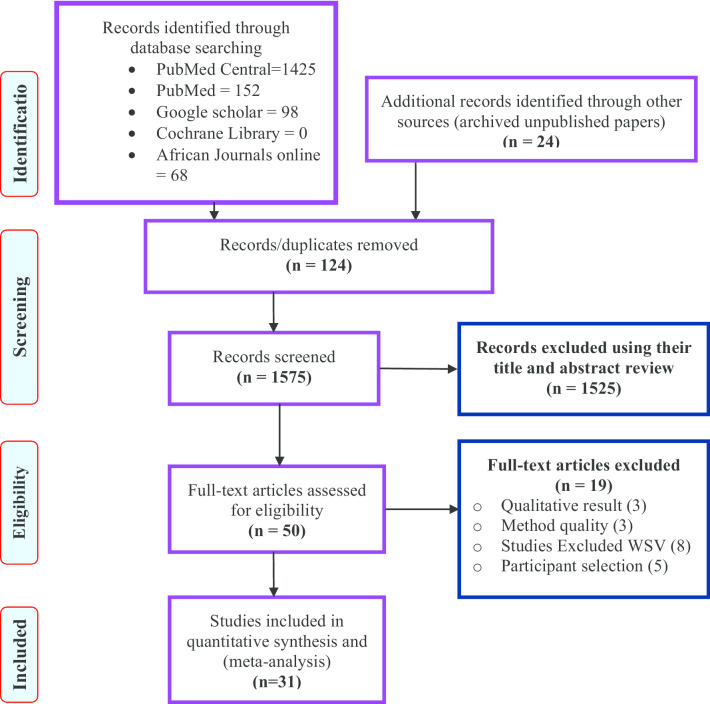
Flow diagram of the included studies in the meta-analysis of workplace sexual violence in Ethiopia

Grey literature deposited at universities and research institutes websites online repository and published studies in Ethiopia were searched. The electronic search of published studies was using PubMed, PubMed Central, MEDLINE, CINAHL, African Journals Online, Cochrane reviews databases and Google Scholar. All studies that reported workplace sexual violence in Ethiopia from July 1998 to June 5, 2020, were included in the review. The core search terms and phrases were "Workplace Violence", "sexual violence", "sex offense", "sexual abuse", "physical violence", "verbal violence", "sexual harassment", and "Ethiopia". The search strategies were developed using different Boolean operators. Notably, to fit the advanced PubMed central database, the following search strategy was applied: [Workplac* Violenc* OR sexual violenc*[MeSH Terms] OR sex offens*[MeSH Terms] OR sexual abus*[MeSH Terms] OR physical violenc* OR verbal violenc* OR sexual harassment AND Ethiopia]. Then we retrieved 1425 articles using this PubMed central searching strategy. To search PubMed, we used the following searching terms with Boolean operators; Workplace AND sexual violence OR physical violence OR verbal Violence OR sexual harassment AND Ethiopia. At this stage, we retrieved 152 articles. The included studies’ references were also retrieved (Additional file 1). Similarly, articles that cited the identified critical articles were observed online (i.e., both ancestor and descendent search strategies were used). We screened the articles using the Preferred Reporting Items for Systematic Reviews and Meta-analyses (PRISMA) statement guidelines [17] (Fig. 1).

### Criteria for the consideration of studies for the review

#### Inclusion criteria

Design: All observational studies that measured the prevalence of workplace sexual violence in Ethiopia were included.

Publication status: All published studies and studies found on websites of Ethiopian universities and research institutes were included.

Language: Due to the feasibility of reading and understanding other languages and reporting articles in other languages is uncommon in Ethiopia. So only articles written in the English language were considered.

Publication or report year: Due to the insufficiency of literature on workplace sexual violence in Ethiopia, we reviewed all research reports from July 1998 until June 5, 2020.

Primary studies scored ≥ 60% of the Joanna Briggs Institute (JBI) criteria for evaluating the quality by two of the authors were included in the meta-analysis [18].

#### Exclusion criteria

Studies that reported sexual violence among refugees, married women, and pregnant women (intimate partner violence) were excluded due to the study’s focus. Similarly, workplace violence studies that did not report workplace sexual violence and did not report workplace sexual violence prevalence or its types were excluded. Additionally, studies that used the workplace to identify participants for violence outside the workplace were excluded. Furthermore, pure qualitative studies assessed workplace sexual violence; reviews, essays, conference abstracts, letters, and commentaries were excluded from the study. Finally, when multiple publications from the same study population within the same year were identified, we included the publication that presented the complete information on results or the publication with the most significant number of cases.

#### Operational definitions

*Workplace sexual violence* is a situation where the employees are abused, threatened, intimidated to have sex or engage in acts of sex without their will in the circumstances related to their work and while commuting to and from work, involved explicit or implicit challenges to their safety, well-being, or health. It includes sexual harassment, attempted rape, and rape [5]. In this study, workplace sexual violence was considered if the primary studies report any type (sexual harassment, attempted rape, or rape). The direct report of workplace sexual violence was found and considered in some studies (Table 1).

**Table 1:** List of the 31 studies and their subcategories included in the meta-analysis of workplace sexual violence in Ethiopia

No	Study	Sample	SV	SH	AR	R	Occupation	Workplaces	Location	Sex
1	Mulugeta et al. [67]	1401	–	137	71	61	Students	High School	Addis Ababa and West Shoa	F
2	Worku and Addise [48]	216	141	–	25	19	Students	High School	Debark	F
3	Tadesse [68]^a^	612	–	256	45	11	Students	University	Addis Ababa	F
4	Fitaw et al. [49]	367	–	162	75	122	Students	High School	Dabat	F
5	Gebreyohannes [45]	1024	352	217	197	57	Students	High School	Mekelle	F
6	Gorfu and Demise [65]	301	–	–	50	61	Students	High School	Jimma Zone	F
7	Arnold et al. [53]	1330	543		102	33	Students	University	Awassa	F
8	Lelisa and Yusuf [69]	377	–	286	–	98	Students	High School	Addis Ababa	F
9	Marsh et al. [54]	387	–	181	–	–	Faculty and Staff	University	Awassa	F
10	Asfaw [55]^a^	516	–	353	118	14	Students	High School	Awassa	F
11	Bekele et al. [64]	764	520	398	–	–	Students	High School	Dire Dawa, Harar, Jigjiga	F
12	Haile et al. [70]	872	–	–	–	38	Students	High School	Addis Ababa	M
13	Shimekaw et al. [50]	536	202	192	–	–	Students	University	Bahir Dar	F
14	Tora [56]	374	–	91	88	42	Students	University	Wolayita Sodo	F
15	Bekele and Deressa [57]	590	–	242	37	12	Students	University	Ambo	F
16	Letta et al. [58]	801	–	–	228	337	Students	High School	Hadiya	F
17	Takele and Setegn [59]	397	–	–	–	27	Students	University	Mada Walabu	F
18	Alemayehu et al. [46]	250	189	150	–	–	CSW	Community	Mekelle	F
19	Bekele et al. [74]	605	–	221	152	66	Students	University	Mada Walabu	F
20	Fute et al. [60]	642	–	84	–	–	Nurses	HF	Awassa	B
21	Jira [71]	203	–	20	–	–	Nurses	HF	Oromia Region	B
22	Mamaru et al. [66]	385	–	348	–	–	Students	University	Jimma Zone	F
23	Mulu et al. [51]	124	–	36	25	16	Students	High School	Debre Markos	F
24	Nimani and Hamdela [61]	332	55	14	4	–	Students	High School	Butajira	F
25	Sendo and Meleku [62]	336	–	–	–	48	Students	University	Awassa	F
26	Adinew and Hagos [5]	473	167	–	–	–	Students	University	Wolayita Sodo	F
27	Abate et al. [72]	435	–	95	–	–	HCW	HF	Addis Ababa	B
28	Amogne et al. [73]	4884	–	–	–	743	CSW	Community	Ethiopia	F
29	Yenealem et al. [52]	531	–	38	–	–	HCW	HF	Gondar Town	B
30	Galu et al. [47]	356	180	–	–	–	Faculty and Staff	University	Mekelle	F
31	Tantu [63]	633	147	–	–	32	Students	University	Wolayita Sodo	F
Total		21,054	2496	3521	1217	1837				

*AR* attempted rape, *R* rape, *CSW* commercial sex workers, *HCW* Health care workers, *HF* health facility, *SH* sexual harassment, *SV* sexual violence, *F* female, *M* male, *B* both sexes

^a^Unpublished studies

*Rape is* any non-consensual penetration of the vagina, penetration obtained by physical body harm, by threatening or deception, or when the victim is unable to give consent [19].

*Attempted rape* is a trial to have sex without consent by coercion, threatening, or deception, or when the victim cannot consent, without the vagina’s actual penetration [20].

*Sexual harassment* is defined as unwanted sexual behaviors, including jokes, verbal comments, and physical contacts intentionally done on women or girls [20].

### Data extraction and quality assessment

We extracted the data using the Joanna Briggs Institute (JBI) tool for cross-sectional studies (Additional file 2) [21]. The tool contains information on study methods, results, and overall study details. The data extraction tool also contains information on study period and year of publication, study area, region, study design and type, sample size, response rate, the prevalence of workplace sexual violence, and prevalence of different forms of workplace sexual violence. All selected articles, after a full review, were appraised using the JBI critical appraisal checklist. The metrics of quality assessment for the included studies were appropriateness of study participants (e.g., sampling frame, sampling procedure, and sample size), study settings, and designs (e.g., description of settings, analysis) as well as the appropriateness of measurements (e.g., validity, reliability). Two authors independently assessed the quality of included articles using the instrument. Any unclear information or disagreements were resolved through discussion. We used the mean quality score to assess the quality of included studies in the meta-analysis [22]. Accordingly, the quality score of the included studies ranged from 6 to 9. The second and third authors were consulted for any discrepancies during the critical appraisal.

### Data analysis

Data were analyzed using comprehensive meta-analysis version 3.0 and MetaXL version 5.3 software. We recalculated the unadjusted prevalence(waited) based on crude numerators and denominators provided by individual studies. The study-specific prevalence variance was stabilized with the Freeman–Tukey double arcsine transformation before pooling the data within a random-effects meta-analysis model. This transformation was to minimize studies with extremely small or extensive prevalence estimates [28]. To assess the presence of publication bias Egger’s asymmetry test was used [23]. A p-value < 0.05 on the Egger test was considered indicative of statistically significant publication bias. Heterogeneity was assessed by the χ^2^ test Cochrane’s Q statistic [24], quantified by H and I^2^ values. The I^2^ statistic estimates the percent of total variation across studies due to actual differences between-study rather than luck. Generally, I^2^ values greater than 60–70% indicate substantial heterogeneity [25]. The source of variation between studies was assessed with subgroup analysis using stratifying variables such as outcome, study location/region, profession, workplace, and sex.

### Publication bias and heterogeneity

**Fig. 2:**
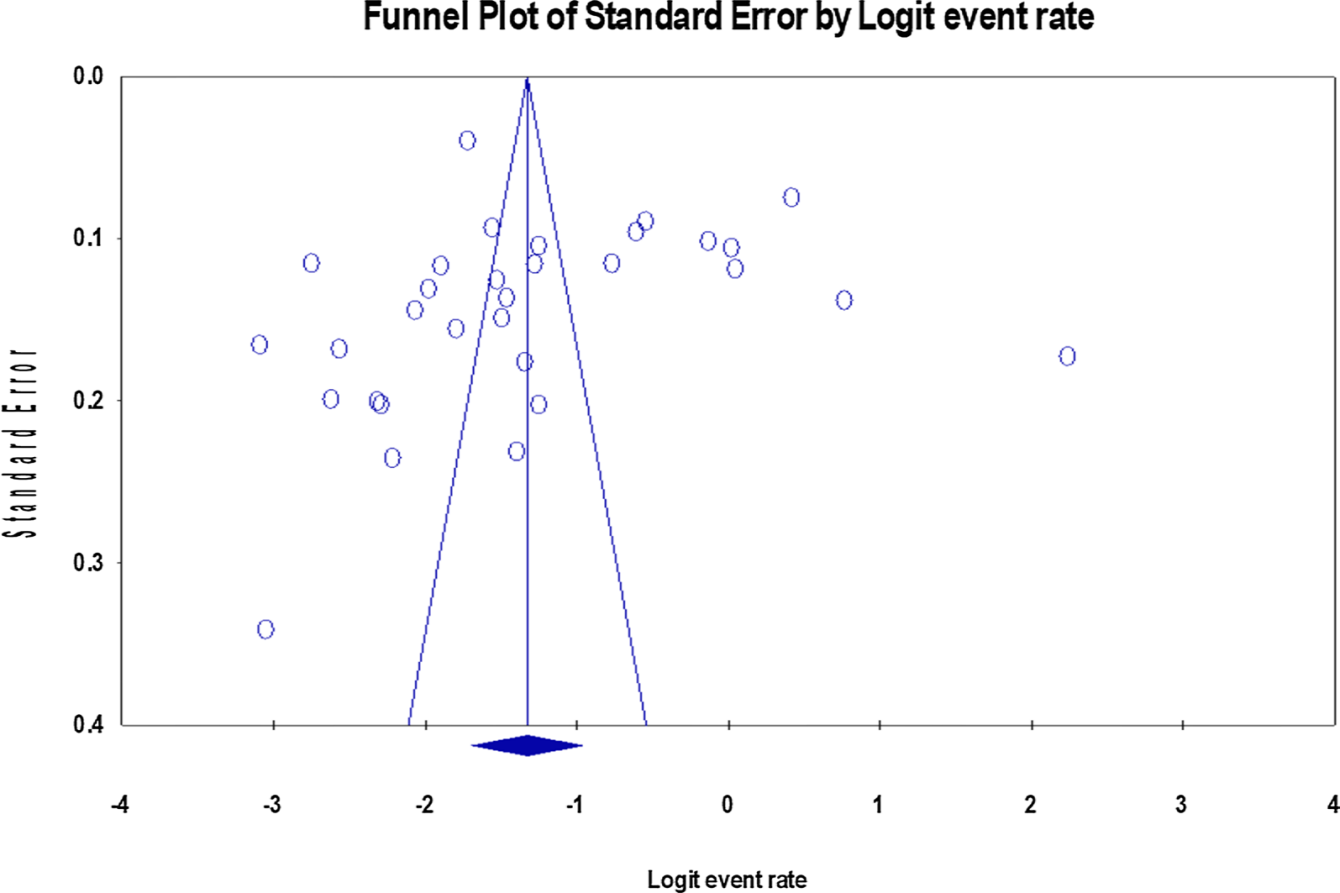
Filled funnel plot of the 31 studies included in the meta-analysis of workplace sexual violence in Ethiopia

The variation in the included studies, heterogeneity, was assessed by visual inspection of the forest plots. The I^2^ statistics and its corresponding p-value were used to determine the statistical significance of heterogeneity. I^2^ statistics of 25%, 50%, and 75% were used to declare low, moderate, and high heterogeneity, respectively [26]. For the valuation of the publication bias of the included studies, we used Funnel plots (Fig. 2). The publication bias’s statistical significance was declared using the Egger regression asymmetry test, setting p < 0.05 [23, 27]. The Duval and Tweedie nonparametric trim and fill analysis using the random-effect analysis was conducted to account for publication bias for meta-analysis results, which showed the presence of publication bias (Egger test, p < 0.05), [28]. Moreover, to identify any possible outlier, sensitivity analysis (by removing each included study at a time) was carried out.

## Results

### Study selection

A total of 165 records (141 using the PUBMED database and 24 additional records identified from advanced google scholar, Cochrane review library, WHO databases, and gray literature from archives of universities in Ethiopia) related to the intended review topic were retrieved. Of these records, 139 studies remained after the removal of 26 duplicated retrievals. Of the 139 studies, 89 studies were excluded because they did not meet the study’s inclusion criteria (JBI). Then, 50 full-text articles/reports were accessed and screened based on the pre-set criteria. Finally, the systematic review and meta-analysis included 31 studies that fulfilled the eligibility criteria (Table 1).

### Study characteristics

Three studies, sexual violence, schooling, and silence, which is teacher narratives from a secondary school in Ethiopia [29], perceived risk factors for gender-based violence among Ethiopian university students [30], and resilience factors, causes and consequences of sexual abuse in housemaids working in Addis Ababa [31] were excluded due to lack of quantitative data for meta-analysis.

Eight studies [32–39] did not include sexual violence as an outcome, three studies [40–42] did not meet the JBI appraisal criteria, and five studies [33–35, 43, 44] were excluded due to the intention of selecting study participants (i.e., studies that base workplace to screen violence but the participants are not directly from the workplaces).

Out of the 31 studies that were eligible and included in the systematic review and meta-analysis, three studies were from north [45–47] and five studies from northwest Ethiopia [48–52]. Twelve studies from South Ethiopia [5, 53–63], one study from South East Ethiopia [64], and two studies from the South West [65, 66] parts of Ethiopia. Six studies from the central part of Ethiopia [67–72], one study on the 11 major cities [73], and one study from the western part of Ethiopia [57].

Regarding the study design, four studies were mixed-method designs, and 27 were cross-sectional. In all studies, 22,176 samples planned, and 21,887 were responded, making an average response rate of 97.62%. The sample size for each included study ranged from 124 in a study conducted in Debremarkos to 4900 in a study conducted among 11 major Ethiopian cities.

### Sensitivity analysis

To identify smaller or larger pooled prevalence reports that could affect the pooled prevalence of sexual violence by giving a wide confidence interval and variance instability, we checked each study’s sensitivity. However, it was found that no study significantly affected the pooled prevalence of sexual violence (Table 2).

**Table 2:** The summary of sensitivity analysis of the included studies

Study	Pooled P (95% CI)	Cochrane Q	p-value	I^2^ (95% CI)
Mulugeta et al. [67]	22.9 (17.1, 29.3)	2960.405	< 0.001	99.020 (98.884, 99.140)
Worku and Addise [48]	22.2 (16.4, 28.6)	3199.319	< 0.001	99.094 (98.971, 99.202)
Tadesse [68]**	22.7 (16.8, 29.2)	3152.117	< 0.001	99.080 (98.954, 99.190)
Fitaw et al. [49]	21.9 (16.1, 28.3)	3167.142	< 0.001	99.084 (98.960, 99.194)
Gebreyohannes [45]	22.4 (16.4, 29.0)	3199.695	< 0.001	99.094 (98.971, 99.202)
Gorfu and Demise [65]	22.3 (16.5, 28.8)	3201.051	< 0.001	99.094 (98.971, 99.202)
Arnold et al. [53]	22.4 (16.4, 29.0)	3200.272	< 0.001	99.094 (98.971, 99.202)
Lelisa and Yusuf [69]	21.4 (15.8, 27.6)	3013.904	< 0.001	99.038 (98.904, 99.155)
Marsh et al. [54]	21.5 (15.9, 27.7)	3054.824	< 0.001	99.051 (98.920, 99.166)
Asfaw [55]**	22.3 (16.4, 28.7)	3199.788	< 0.001	99.094 (98.971, 99.202)
Bekele et al. [64]	21.1 (15.9, 26.9)	2584.692	< 0.001	98.878 (98.714, 99.021)
Haile et al. [70]	23.0 (17.2, 29.4)	2996.146	< 0.001	99.032 (98.898, 99.150)
Shimekaw et al. [50]	21.8 (16.0, 28.1)	3110.590	< 0.001	99.068 (98.940, 99.180)
Tora [56]	22.3 (16.5, 28.8)	3201.074	< 0.001	99.094 (98.971, 99.202)
Bekele and Deressa [57]	22.7 (16.8, 29.2)	3151.395	< 0.001	99.080 (98.954, 99.190)
Letta et al. [58]	22.6 (16.7, 29.1)	3173.202	< 0.001	99.086 (98.962, 99.196)
Takele and Setegn [59]	22.8 (17.0, 29.3)	3146.895	< 0.001	99.078 (98.953, 99.189)
Alemayehu et al. [46]	21.6 (15.9, 27.9)	3128.377	< 0.001	99.073 (98.946, 99.185)
Bekele et al. [74]	22.2 (16.3, 28.7)	3196.346	< 0.001	99.093 (98.970, 99.201)
Fute et al. [60]	22.6 (16.7, 29.1)	3185.006	< 0.001	99.089 (98.966, 99.198)
Jira [71]	22.7 (16.8, 29.2)	3187.969	< 0.001	99.090 (98.967, 99.199)
Mamaru et al. [66]	20.2 (15.5, 25.3)	2183.174	< 0.001	98.972 (98.465, 98.851)
Mulu et al. [51]	22.3 (16.5, 28.8)	3200.941	< 0.001	99.094 (98.971, 99.202)
Nimani and Hamdela [61]	23.0 (17.1, 29.4)	3128.856	< 0.001	99.073 (98.946, 99.185)
Sendo and Meleku [62]	22.5 (16.6, 29.0)	3196.198	< 0.001	99.093 (98.969, 99.201)
Adinew and Hagos [5]	21.8 (16.1, 28.2)	3132.768	< 0.001	99.074 (98.948, 99.186)
Abate et al. [72]	22.2 (16.4, 28.7)	3198.478	< 0.001	99.093 (98.970, 99.202)
Amogne et al. [73]	22.4 (16.0, 29.6)	3142.350	< 0.001	99.077 (98.951, 99.188)
Yenealem et al. [52]	22.8 (17.0, 29.3)	3133.164	< 0.001	99.074 (98.948, 99.186)
Galu et al. [47]	21.4 (15.8, 27.6)	3031.239	< 0.001	99.043 (98.911, 99.160)
Tantu [63]	22.6 (16.7, 29.1)	3171.915	< 0.001	99.086 (98.961, 99.195)

**Unpublished studies

### The pooled prevalence of workplace sexual violence

**Fig. 3:**
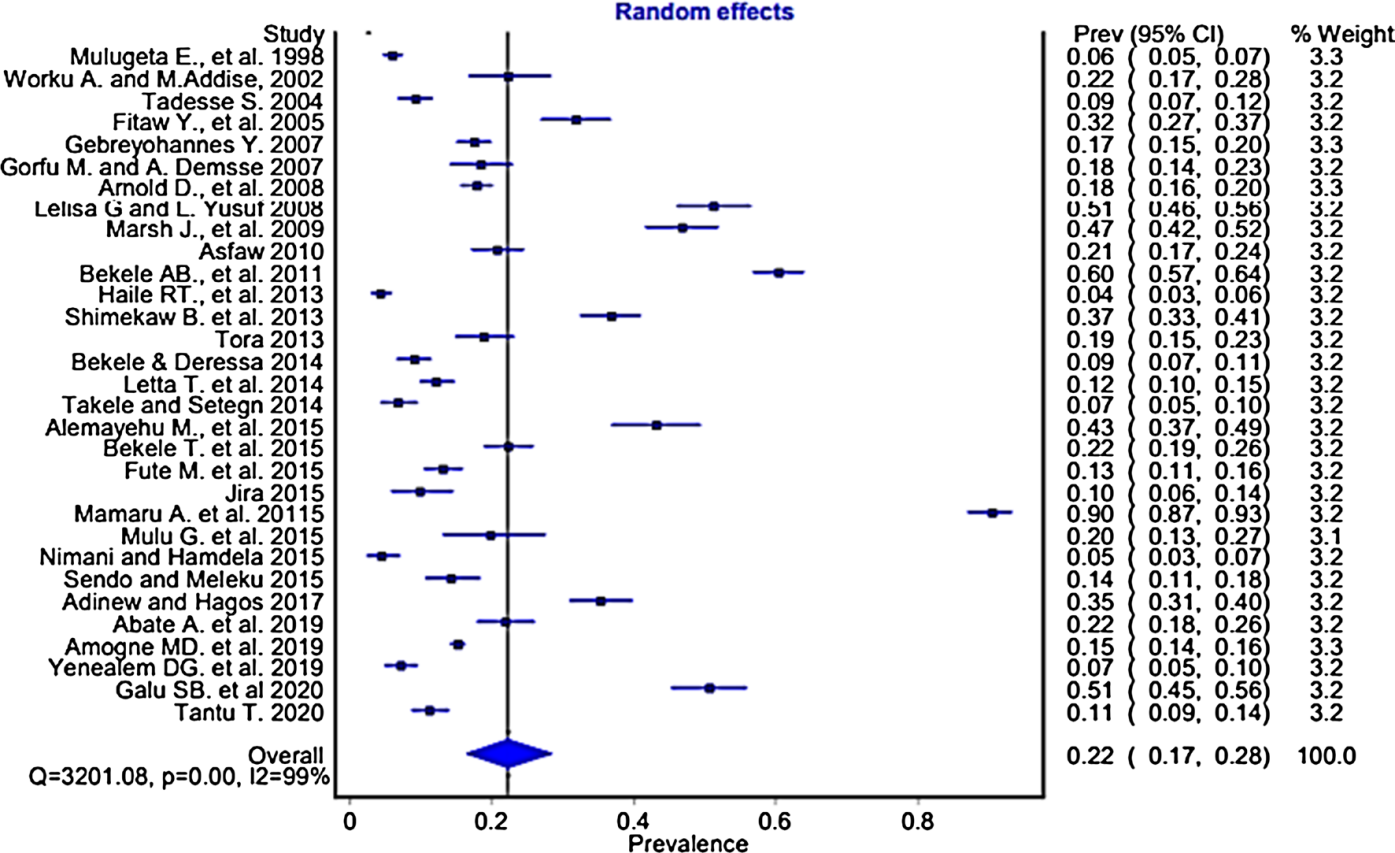
Forest plot of the prevalence of workplace sexual violence in Ethiopian workplaces

Based on the random effect model, the overall pooled prevalence of WSV among the 21,054 respondents with a mean age of 22.22 (± 3.15) years after combining each study outcome is 22% (95% CI 17, 28%). The heterogeneity test of included studies showed significant heterogeneity, I^2^ = 99% and p < 0.001 (Fig. 3). Thus, subgroup analyses were conducted using occupation/profession, type of violence, workplace, and sex.

### Sub-group analysis

**Fig. 4:**
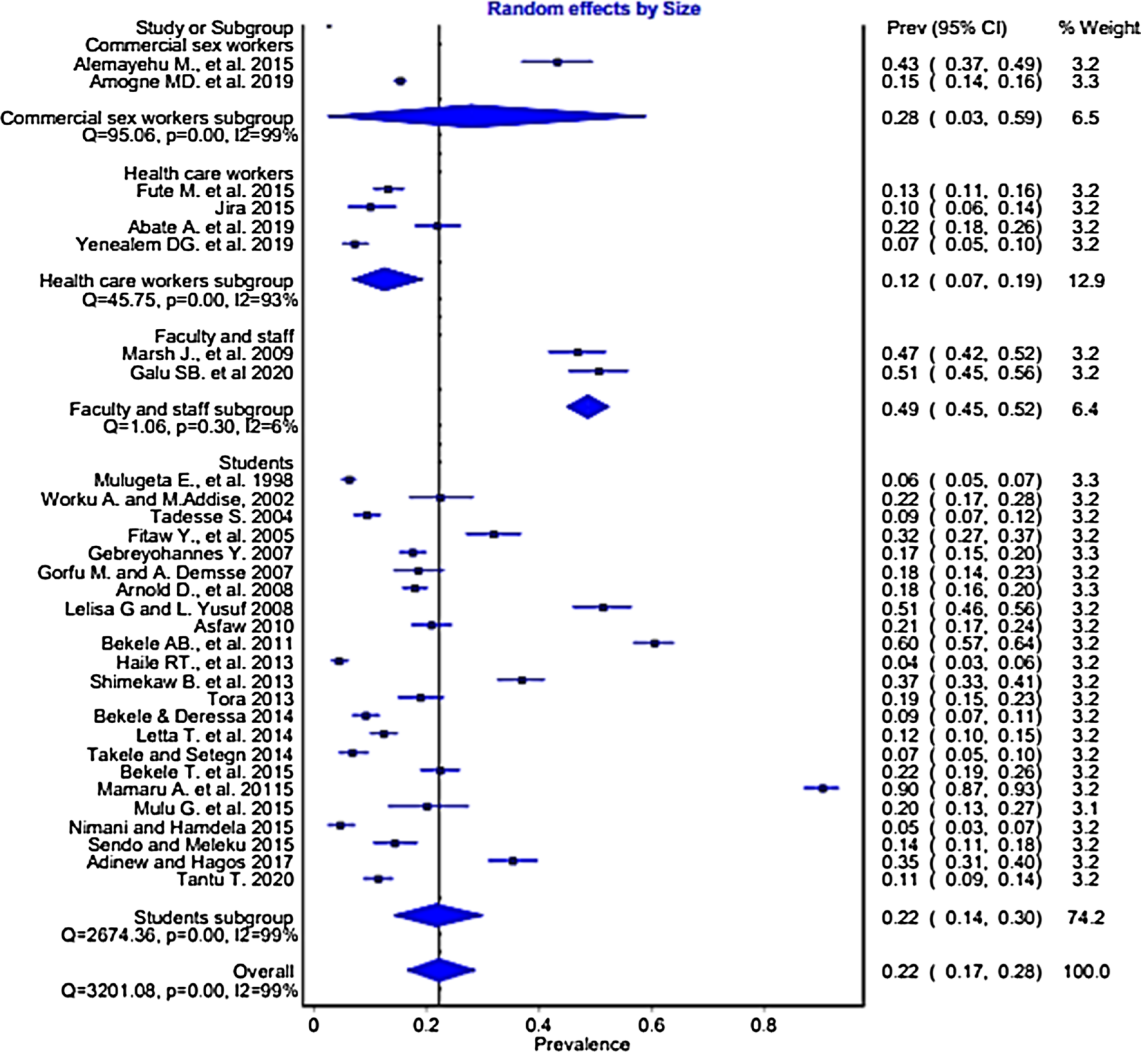
Forest plot of the pooled prevalence of workplace sexual violence in Ethiopian workplaces

After realizing the heterogeneity of the studies, subgroup analysis was conducted based on different characteristics. The subgroup analysis by profession showed the highest prevalence among female university staff (both academic and supportive) 49% (95% CI 45, 56%), followed by CSW 28% (95% CI 3, 59%) (Fig. 4). Similarly, the pooled prevalence was 14.1% (95% CI 10.4, 18.9%, p < 0.001) for attempted rape, 8% (95% CI 5.7, 11.1%, p < 0.001) for rape, and 33.2% (95% CI 24.1, 43.7%, p = 0.002) for SH (Table 3).

**Table 3:** Stratified analysis of the 31 studies included a meta-analysis based on the outcome, sex, and Ethiopian regions

Stratifying variable	Sample size	Fixed effects P (95%CI)	Random effects P (95%)
Outcomes			
Attempted rape	1217	0.183 (0.174, 0.193)	0.141 (0.104, 0.189)
Completed rape	1837	0.124 (0.119, 0.130)	0.080 (0.057, 0.111)
Sexual harassment	3521	0.364 (0.354, 0.375)	0.332 (0.241, 0.437)
Sex			
Both	1811	0.141 (0.125, 0.158)	0.122 (0.074, 0.196)
Female	18,371	0.277 (0.272, 0.283)	0.209 (0.168, 0.257)
Male (1 study)	872	0.044 (0.032, 0.059)	0.044 (0.032, 0.059)
Location			
Tigray	1630	0.276 (0.034, 0.567)	0.361 (0.128, 0.615)
Amhara	1774	0.226 (0.088, 0.381)	0.226 (0.100, 0.368)
Oromia	2481	0.238 (0.007, 0.538)	0.241 (0.017, 0.523)
SNNPR	5787	0.177 (0.110, 0.249)	0.182 (0.122, 0.249)
Addis Ababa	2296	0.142 (0.000, 0.345)	0.188 (0.022, 0.400)
Cross region	7048	0.169 (0.000, 0.499)	0.239 (0.020, 0.515)

**Fig. 5:**
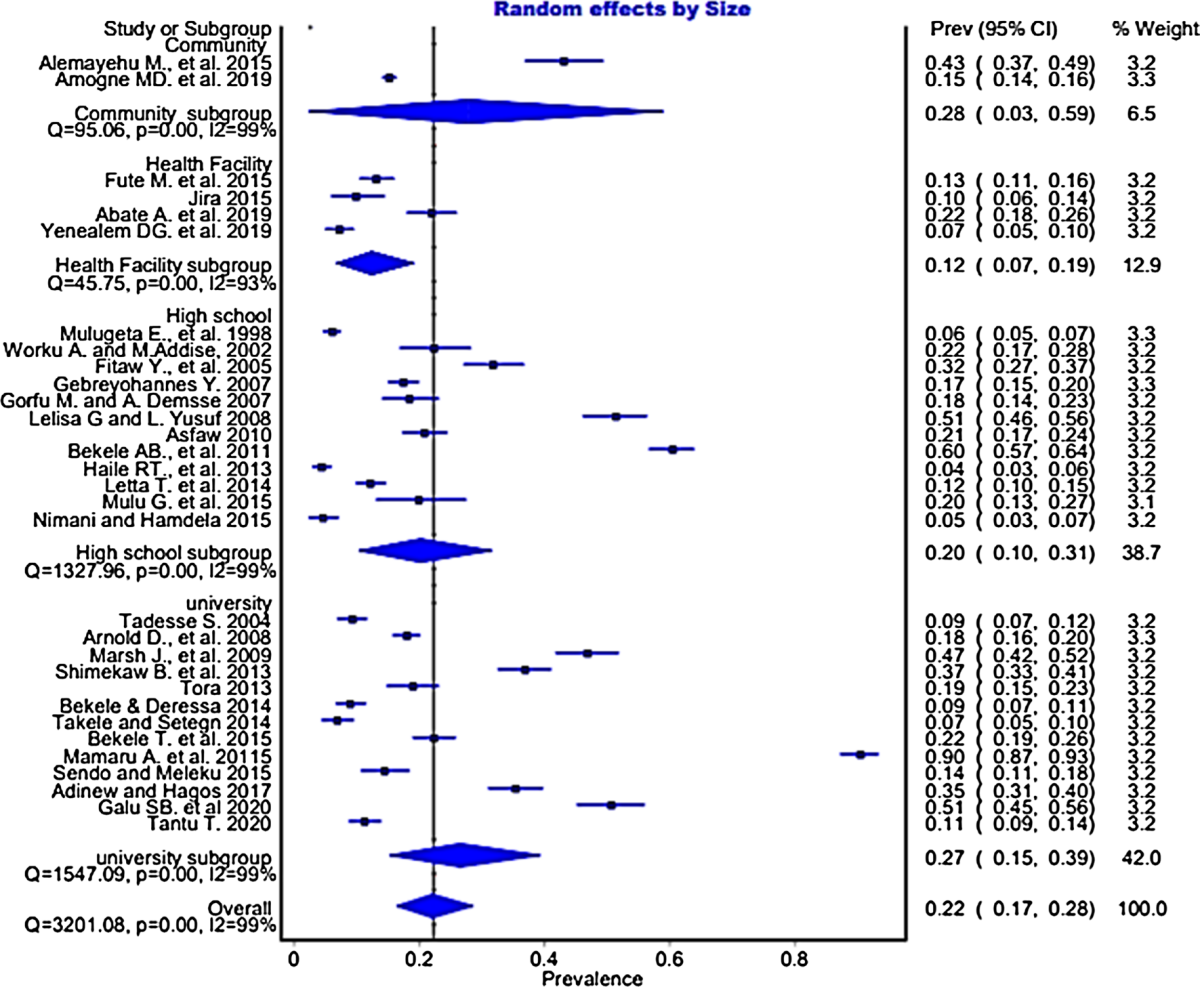
Forest plot of the pooled prevalence of workplace sexual violence in Ethiopian workplaces

Based on study location, Tigray national regional state had the highest prevalence of pooled WSV 36.1% (95% CI 12.8% to 61.5%), followed by Oromia national regional state 24.1% (95%CI 1.7% to 52.3%), Amhara national regional state 22.6% (95% CI 10% to 36.8%), Addis Ababa 18.8% (95% CI 2.2% to 40%), and SNNPR 18.2% (95% CI 12.2% to 24.9%). The pooled prevalence of cross-regional studies was 23.9% (95% CI 2% to 51.5%). The pooled prevalence of WSV was 20.9% (95% CI 16.8% to 25.7%) among studies that reported SV among females only, and 12.2% (95% CI 7.4% to 19.6%) among studies that reported SV in both sexes (Table 3). On the other hand, the pooled WSV prevalence among female CSW 28%(95% CI 3,59%), and university students 27%(95% CI 15,39%) were the highest (Fig. 5).

## Discussion

This systematic review and meta-analysis indicated that the pooled prevalence of workplace sexual violence in Ethiopia was 22% ranging from 17 to 28%. This finding was higher than the prevalence of workplace violence among Korean employees [75] and American employees [76]. However, it was lower than workplace sexual violence among Nigerian employees (63.8%) [77]. The differences in definitions and classifications used, differences in methodologies for collecting and processing information, differences in time frames analyzed, and differences in culture in the experience of violence and harassment might delimit the concept of WSV. So, it is challenging to compare statistics on exposure to workplace sexual violence across different countries. The difference might be due to the difference in workplaces [78] in Ethiopia and the countries mentioned above. The countries mentioned above, including Ethiopia, prohibited workplace sexual violence. So, the difference in the prevalence of WSV between Korea, the USA, Nigeria, and Ethiopia are probably not due to Anti-violence (harassment) laws. This study might be different due to methodological, setting, population difference, development status, and law enforcement between Korea, the USA, and Ethiopia. We encountered one challenge while conducting this study because more primary studies were conducted among educational settings, and few on university staff, commercial sex workers, nurses, and general health care workers. This finding indicates that the concept of workplace violence is entirely new in Ethiopian society, and there are no regulations or policy interventions specific to violence in the workplace. This result may result in reduced sensitivity to the perception of workplace violence in Ethiopia.

The sub-group analysis of WSV by type in our study indicated that the pooled prevalence was 14.1%, 8%, and 33.2% for attempted rape, rape, and sexual harassment, respectively. These findings were lower than the Nigerian study [77] and higher than the Korean study [75]. The differences in the prevalence estimates of sexual violence types might be due to the difference in method. Though it reports all forms of workplace sexual violence, the Nigerian national study was cross-sectional with a small sample of participants from different professions. However, ours is a systematic review and meta-analysis with a large sample of participants. This systematic review and meta-analysis finding implies that legislation and prohibitions of only sexual harassment might not be adequate to address all the forms of WSV occurring in the Ethiopian workplace context. Thus, we suggest the necessity for better protection actions against workplace sexual violence to create a safe working environment for Ethiopian employees. We also would like to recommend all workplaces to implement the proclamation set by the Ethiopian ministry of labor and social affairs.

It also indicated that the pooled prevalence of workplace sexual violence was high among female employees (20.9%). The finding was not in line with the previous systematic review [79]. However, the previous study finding was focused on the physical violence of workplaces. The subgroup analysis also indicated that the pooled prevalence of workplace sexual violence among health care professionals was 12% (95% CI 7% to 19%), which is similar to the previous systematic review and meta-analysis [80]. This finding is higher than the workplace sexual violence in America [76] and China [81]. One of the crucial reasons for the study differences might be the difference in the measurement of sexual violence, sociodemographic status, culture, and study methods.

At the same time, workplace sexual violence among commercial sex workers was 28% (95% CI 3% to 59%), which is consistent with the previous global systematic review [82] that ranged from 32 to 55%, studies in Mexican cities (11.7%) [83], and with a study conducted in Northern Uganda (49%) [84]. This finding was similar due to the similarity of socioeconomic characteristics, work context, social and behavioral characteristics of female commercial sex workers. In the meantime, our study’s findings share common problems of LMICs, including Uganda and Mexico. It implies that there could be poverty, childhood abuse history, police abuse, high rates of STIs [85], and high rates of drug and alcohol use [86–91], which needs the development of evidence-based HIV/STI prevention programs and treatment services for this high-risk population [84].

In line with a recent global systematic review among staff in higher education [92], the pooled prevalence of workplace sexual violence among female faculty, including the staff, was high in this study, 49% ranging from 45 to 56%. This study finding is similar to the study findings in Nepal (53.8%) [93] and Malaysia (52.7%) [94]. It is slightly higher than the finding of the study conducted in Lebanon (41.9%) [95]. It is also lower than the study conducted in America (68%) [96]. The difference might be considering any action against women as sexual Violence in Ethiopian studies and the variations in socioeconomic status, sociodemographic variables of the study participants, and sample size. However, there is still no adequate information on the prevalence and resulting health effects of workplace sexual violence among female faculty and staff in the academic settings of LMICs. Therefore, there is no adequate knowledge to draw from and clarify tendencies in the data.

Similarly, the sub-group analysis using the profession in this study indicated that the pooled workplace sexual violence was 22% among students. Specific to the education settings’ location, the pooled prevalence was 20% in high school and 27% in universities. In this systematic review and meta-analysis, more primary studies were included from education settings than other workplaces (23 studies, 13 at universities and ten at high school level), which indicated that more primary studies were conducted in education settings in Ethiopia. This systematic review and meta-analysis study identified a discrepancy of sexual violence prevalence from study to study (6% [61] to 90% [66]). This finding was similar to the previous global systematic review finding [97]. This study’s findings conclude that sexual violence in education centers (both in high school and Universities) is a considerable concern in Ethiopia. Thus, in addition to the need to conduct longitudinal cohorts and more comprehensive follow-up studies, this study suggests the design of suitable preventive measures, development of inter-cultural research projects involving different regions, and consideration of social norms, sense of community, pro-social modeling, organizational policies, and the physical environment of educational centers’ beyond the individual, group, and situational levels for future researchers in line with a previous systematic review [98].

Furthermore, in the sub-group analysis based on regional states, the pooled prevalence of workplace sexual violence was high in the Tigray regional state (36.1%) and low in Addis Ababa city (18.8%) and SNNPR (18.2%) region. However, it was almost similar in the Amhara National Regional State (22.6%), in the Oromia National Regional State (24.1%), and cross-regional studies (23.9%). The low prevalence estimates in the Addis Ababa city and SNNPR region could be due to better awareness of sexual violence practices in workplaces. The high prevalence from the Tigray region could be because a study with high prevalence among commercial sex workers and female administrative staff was included in the study that might affect the overall pooled prevalence estimate in that area. Besides, the studies conducted in Tigray’s regional state were only from a single city (Mekelle). Thus, this study suggests that researchers conduct more studies in different parts of the region and suggest the region consider implementing gender policy and rules and regulations that safeguard employees from sexual violence. Moreover, there is a need to develop interventions that could empower women employees, control and monitor the law’s implementation, create awareness for men, and develop organizational anti-sexual violence policies.

The critical policy implication of this study findings relies on the prevention and control of workplace sexual violence. These findings suggest that it is essential to implement strategies to reduce workplace sexual violence prevalence over different workplaces. Similar to the recommended strategies suggested to reduce general workplace violence, adequate staffing, and education and training programs are essential to assist employees and students. Similarly, public awareness creates workplace sexual violence negativity through a mass media campaign, enforcing appropriate policies and legislation, such as encouraging staff to report such acts and judicial punishment on the perpetrators promptly. It is mandatory to apply preventive strategies urgently among workplaces, particularly the universities, to reduce the negative impacts of workplace sexual violence among employees. This study’s finding is an essential input for governmental organizations (Ministry of Health, Ministry of social and labor affairs, Ministry of women and child, general attorney), non-governmental organizations (both local and international), and other relevant stakeholders such as civic societies who want to work on limiting and reducing workplace sexual violence. However, there is a need to conduct more primary studies in other professions and workplaces, such as hospitality workplaces, industrial parks, and factories, that leads the world’s workplace sexual violence report.

We need to mention some potential limitations of this systematic review and meta-analysis. Although the number of primary studies for estimating workplace sexual violence prevalence was adequate, it was more among education settings than other jobs. Second, due to the differences in associated factors based on the profession and workplaces, this study did not include the review of associated factors. Third, this study was the first to estimate the pooled prevalence of any WSV and incorporated all types of WSV in Ethiopia; however, the primary studies on sexual violence were conducted on limited professions (see Table 1).

## Conclusions

This review and meta-analysis indicated that the prevalence of sexual violence in Ethiopian workplaces is high. Of the types of workplace sexual violence, workplace sexual harassment is high. It is also exceptionally high among female university staff, commercial sex workers, and Tigray regional state workplaces. Governmental organizations, non-governmental organizations, and other stakeholders should develop effective programs and interventions to reduce workplace sexual violence prevalence over different (students, commercial sex workers, nurses, health care workers, university staffs) Ethiopian workplaces. Also, the prevention of sexual violence in the workplace is possible but cannot be fully realized without understanding the problem. Therefore, conducting more primary studies of workplace sexual violence in different workplace settings, particularly in hospitality workplaces, which leads the report of WSV worldwide, using nationally representative data is essential to understand this problem’s potential magnitude and the most common forms of sexual violence perpetrated to inform prevention efforts. Also, examining the impacts of these forms of violence provides some additional context and uncovers the adverse health effects this kind of sexual violence has on victims. Moreover, bringing attention to and better contextualizing these workplace sexual violence experiences would increase the Ethiopian context’s ability to prevent them.

## Supplementary Information

**Additional file 1**. Database searching strategies.

**Additional file 2.** Joanna Briggs Institute (JBI) tool for appraisal of cross-sectional studies.

## Data Availability

All data generated or analyzed are included in the results of the document.

## Abbreviations

AR: Attempted rape
CI: Confidence interval
CMA: Comprehensive meta-analysis
CR: Completed rape
CSW: Commercial sex workers
HCW: Health care workers
HF: Health facility
ILO: International Labor Organization
JBI: Joanna Briggs Institute
PRISMA: Preferred Reporting Items for Systematic Reviews and Meta-analyses
SH: Sexual harassment
SNNPR: South nations and nationalities of people region
SPSS: Statistical package for social sciences
SV: Sexual violence
UN: United Nations
WSV: Workplace sexual violence
