# Supplementary material for: Prevalence of sexual violence in Ethiopian workplaces: systematic review and meta-analysis
Source: Reprod Health. 2020 Dec 9;17:195. doi: 10.1186/s12978-020-01050-2 (PMC7724841; doi:10.1186/s12978-020-01050-2)
Supplement: Supplementary file 1 — Additional file 1. Database searching strategies. [file 12978_2020_1050_MOESM1_ESM.docx]

| **Query** | **Items found** |
| --- | --- |
| **PubMed Central** | |
| (((((((Workplac* Violenc*) OR sexual violenc*[MeSH Terms]) OR sex offens*[MeSH Terms]) OR sexual abus*[MeSH Terms]) OR physical violenc*) OR verbal violenc*) OR sexual harassment) AND Ethiopia | 1425 |
| workplace sexual violence | 3355 |
| (((((Workplac* violence) OR workplac* sexual violenc*) OR Physical violenc*) OR verbal violenc*) OR sexual harassment) AND Ethiopia | 849 |
| workplac* violence OR workplac* sexual Violenc* AND Ethiopia | 89 |
| Workplac* AND sexual violenc* OR attempted rap* OR rap* AND Ethiopia | 208 |
| Workplac* OR sexual violenc* OR attempted rap* OR rap* AND Ethiopia | 3196 |
| Workplac* AND sexual Violenc* OR attempted rap* OR rap* AND Ethiopia | 208 |
| Workplac* AND sexual Violenc* OR physical Violenc* OR verbal Violenc* OR sexual harassment AND Ethiopia | 849 |
| Workplace AND sexual Violence OR physical Violence OR verbal Violence OR sexual harassment AND Ethiopia | 4327 |
| workplac* violence OR workplac* sexual Violence AND Ethiopia | 278 |
| workpla* violence OR workplac* sexual Violence AND Ethiopia | 278 |
| workplace violence AND Ethiopia | 361 |
| **PubMed** | |
| Workplace AND sexual Violence OR physical Violence OR verbal Violence OR sexual harassment AND Ethiopia | 152 |
| Workplac* AND sexual violenc* OR physical violenc* OR verbal violenc* OR sexual harassment AND Ethiopia | 118 |
| **Google scholar** | |
| Workplac* Violenc* OR sexual violenc*[MeSH Terms] OR sex offens*[MeSH Terms] OR sexual abus*[MeSH Terms] OR physical violenc* OR verbal violenc* OR sexual harassment AND Ethiopia | 98 |
| Cochrane Library | |
| Workplac* Violenc* OR sexual violenc*[MeSH Terms] OR sex offens*[MeSH Terms] OR sexual abus*[MeSH Terms] OR physical violenc* OR verbal violenc* OR sexual harassment AND Ethiopia in Title Abstract Keyword | 0 |
| **Medline** | |
| Sexual violence AND Ethiopia | 217 |
| Workplace AND sexual violence AND Ethiopia | 56 |
| **African Journals Online** | |
| Workplace Violence OR sexual violence OR sex offense OR sexual abuse OR physical violence OR verbal violence OR sexual harassment AND Ethiopia | 68 |
